# Flow dynamics

**DOI:** 10.1186/s13049-024-01232-y

**Published:** 2024-06-27

**Authors:** Thomas Schmutz, Christophe Le Terrier, Vincent Ribordy

**Affiliations:** 1 Service des urgences-SMUR, Fribourg Cantonal Hospital (HFR), Fribourg, 1708 Switzerland; 2 Service des urgences-SMUR, Fribourg Cantonal Hospital (HFR), Chemin des Pensionnats 2/6, Villars-sur-Glâne, 1752 Switzerland

The impact of overcrowding in emergency departments (ED) has been identified as leading to an increase in patient mortality and, ultimately, burnout among health professionals working in this setting. This problem now affects many European countries, including Switzerland [1]. The causes of ED embolization and patient flow obstruction are numerous. However, the current focus is on the overcrowding of premises and overstretching of teams due to patients lying on stretchers waiting for hospitalisation or referral due to a lack of available beds (boarding). A recent study in France highlighted the excess mortality rate among elderly patients who spend a night in the ED because they cannot be admitted to hospital [2]. Based on these results, the President of the SAMU-Urgences de France (emergency services) is attempting to prompt a political response from the French government to address this issue [3]. Similarly, the President of the European Society for Emergency Medicine (EuSEM) in Belgium has expressed his concern regarding patient flow to EDs this year [4] and considers that the participation of emergency physicians in regulating patient flow and adjusting staffing levels are necessary.

Several strategies have already been proposed to alleviate the bottleneck of patient flow downstream of the ED. Some have a significant impact on patient boarding, such as smoothing elective admissions and surgeries, redistributing inpatient service beds, and temporary boarding in inpatient corridors [5]. Due to economic constraints, increasing the number of hospital beds is not always feasible, making the implementation of hospital bed management structures a necessity. This concept is not new. It originated in Anglo-Saxon countries and is now becoming widespread in many European hospitals. The aim is to optimize the supply of hospital beds, while maintaining very high occupancy rates for profitability purposes. In France, the pace of implementation accelerated around 10 years ago following national recommendations and two out of three hospitals now have a bed management unit [6, 7]. However, despite this, French EDs have not been able to contain the problem. In 2018, 100,000 French patients spent the night in the ED due to a shortage of available beds [2]. Unfortunately, the situation has not improved since then and the problem is being exacerbated by a shortage of medical and nursing staff. Switzerland is also beginning to experience similar difficulties, with a lack of bed reserves rendering bed management units inoperable for the same reasons. At present, several of Switzerland’s public cantonal hospitals are facing financial difficulties. As a result, they are being forced to implement cost-cutting measures, restructure their operations, and closely monitor wage expenses.

In the canton of Fribourg, the COVID-19 pandemic and the closure of several EDs due to economic reasons have caused overcrowding in the canton’s sole ED and overloaded the hospital. To manage this situation, and in the absence of a reserve of valuable beds, the bed management unit has evolved into a flow management unit. The aim of this initiative is to offer a more appropriate response to the flow problem and enhance the efficiency of the medical, nursing and administrative processes for each patient from admission to discharge. The flow management unit reports directly to the general hospital directorate and collaborates closely with the medical and nursing directorates. The hospital has the authority to assign patients to departments other than their specialty, pause scheduled activities, or even use a corridor bed in any department if no other beds are available. This decision is significant because it prevents patients from being exclusively housed in the ED. The flow of patients is well-coordinated, with a steering committee led by the hospital director who meets every Monday with department heads from across the hospital group to discuss flow management. The hospital’s occupancy rate is regularly assessed and decisions are made in the case of capacity overload. At the end of each week, when the hospital is under pressure, a meeting is held to make necessary decisions. These decisions, supported by the medical and nursing directorates, are communicated throughout the establishment to ensure that patients are discharged without any obstacles. The senior emergency physician in the ED is responsible for regulating the flow of patients. Notably, the hospital has now managed to avoid patients waiting in corridors on stretchers due to a lack of inpatient beds [8]. However, despite high occupancy rates, the implementation of corridor beds within hospital departments has been sporadic.

Our daily commitement pay off [4]. The impact on patient care conditions is significant, and the absence of patients in the corridors of the ED greatly improves working conditions and care. These results were achieved while maintaining intense pressure on teams throughout the hospital. To support them, new hospital jobs were created, including case bed-flow and capacity manager positions, all overseen by a chief operating officer and business analysts. Efforts are being made to ensure an efficient patient flow in order to free up precious beds due to the lack of available reserves.

However, it is important to note that the situation is not entirely positive. The system is being pushed to its limits and there is limited room for manoeuvre. Paradoxically, the need for additional medical and nursing staff to keep up with the pace is restricted due to the inability to achieve financial equilibrium. Some care workers are exhausted and leaving their jobs, while younger doctors are struggling to find their place in a hospital that prioritises profitability over patient care [9]. It is important for politicians to acknowledge this reality. The absence of patients on stretchers in emergency corridors is a reflection of the hard work of the entire hospital staff. This indicator of hospital quality should be valued by economic and regulatory incentives as a means to adapt resources to flow. It is also important to maintain objectivity and avoid subjective evaluations as failure to do so may result in a new overflow.
